# Three dimensional printed wound dressings: recent progresses

**DOI:** 10.1097/JS9.0000000000000129

**Published:** 2023-03-03

**Authors:** Hitesh Chopra, Priyanka, Om Prakash Choudhary, Talha B. Emran

**Affiliations:** a Chitkara College of Pharmacy, Chitkara University, Punjab, India; b Department of Veterinary Microbiology, College of Veterinary Science, Guru Angad Dev Veterinary and Animal Sciences University (GADVASU), Rampura Phul, Bathinda, Punjab, India; c Department of Veterinary Anatomy and Histology, College of Veterinary Sciences and Animal Husbandry, Central Agricultural University (I), Selesih, Aizawl, Mizoram, India; d Department of Pharmacy, BGC Trust University Bangladesh; e Department of Pharmacy, Faculty of Allied Health Sciences, Daffodil International University, Dhaka, Bangladesh

Highlights

- Trauma and pathophysiological problems have made skin wounds.
- Skin has a greater capacity for regeneration than most other tissues.
- Chronic wounds are expensive to treat since they require a number of different procedures.
- Three-dimensional printing can be a boon to wound treatment.

The prevalence of trauma and pathophysiological problems has made skin wounds a major problem in modern medicine. Hemostasis, inflammation, proliferation, and extracellular matrix remodeling are only a few of the molecular activities involved in a typical wound healing process that are tightly synchronized and controlled.^1^,^2^ Extreme skin loss from large trauma wounds does not result in skin regeneration. This is the case whether the lesion was caused by an accident, a burn, or a disease such as obesity or type II diabetes. Although skin has a greater capacity for regeneration than most other tissues, scarring is the usual method of treatment for extensive, serious wounds. Burns, diabetes, and pressure ulcers are just a few examples of the chronic wounds that put a heavy financial and emotional strain on patients, medical staff, and governments worldwide.^3^,^4^ Chronic wounds are highly expensive to treat since they require a number of different procedures. It is important to get wounds treated quickly so that they do not become worse and cause further tissue damage and hypertrophic scarring. Major scarring can occur in individuals who get inefficient or delayed treatments, which can cause permanent problems like deformity and mobility loss. Loss of hair, glands, skin, and even the circulatory system or tissue death can be avoided with prompt repair. The quality of a patient’s life is greatly improved by effective wound care since it not only speeds healing but also improves the patient’s ability to eat and sleep.

An unmet medical need led to the development of advanced multipurpose wound dressings. Once a wound has reached a certain diameter, it cannot heal on its own. Furthermore, in some people, the wound becomes permanently damaged, which can be fatal. Currently, the finest procedures for wound therapy are referred to as the ‘gold standard.’ Dermal replacements, skin expansion procedures, and flap grafts are all examples.^5^ The lack of donor sites and the subsequent risk of malfunction or psychological distress due to the formation of hypertrophic scars or keloids are the main obstacles to the widespread use of these procedures. Another problem that needs fixing is that not all regions have access to the same technologies. As a result, coming up with innovative answers to these problems is crucial. It has been previously reported that when traditional wound dressings are applied to a wound, they soak up the wound exudates and dry up, causing the surrounding tissues to peel away and potentially exposing the lesion to more infection. Low oxygen permeability, a lack of biomimicry, and inadequate medication loading are further problems associated with conventional wound dressings. Technologies developed through tissue regeneration engineering to replace skin provide desirable solutions to these drawbacks.

We may be able to get over these obstacles to effective wound dressing by combining three-dimensional (3D) printing technology with biocompatible hydrogels.^6^ Subtle microcomponent design using 3D printing technology can control the release of various biologically useful substances. Hydrogels may be loaded with a wide variety of materials, including antimicrobial nanoparticles and other biological compounds, before being used in printing processes. The most notable benefits of using 3D printing technology to create hydrogel wound patches are their great dependability and low cost. Conventional ways of making things, like molding, casting, forming, and machining, work well for high volumes but not for complicated designs that use more than one material.^7^

Bioinks are the raw materials for 3D printing artificial, living tissue. Bioprinting of mechanically stable constructs of varying architectural styles is made possible by the bioinks’ adaptable composition via a wide range of materials. Materials that are used to create bioinks can be either natural or synthetic. These substances, also known as biopolymers, are biocompatible and biodegradable. Antibiotic active substances, or antimicrobial peptides, are used in wound healing applications, and they are frequently combined with growth factors to stimulate cell growth, proliferation, and migration.

Long *et al*.^8^ developed a 3D-printed chitosan (CS)-pectin (PEC) hydrogel for the delivery of lidocaine hydrochloride. Hydrogels were made through the physical crosslinking of CS and PEC polysaccharides. Lyophilization followed the 3D printing of the scaffolds on an extrusion-based 3D printer with a mechanical positive displacement dispensing system. The hydrogels printed in 3D had high levels of printability, dimensional integrity, and skin adhesion. 3D-printed hydrogels are well suited for absorbing exudates and keeping a moist wound healing environment, as evidenced by their high swelling ratio and water absorption. The bioadhesion strength of the 3D-printed dressings, in the range of 86.5–126.9 g, was comparable to that of commercially available wound dressings. All formulations demonstrated rapid but sustained lidocaine hydrochloride release over a 5-hour *in vitro* evaluation.

Muwaffak *et al*.^9^ prepared patient specific wound dressings using polycaprolactone-based filaments and entrapping metal ions such as zinc, copper, and silver, the presence of metallic ions helps in fighting against antimicrobial resistance. The wound dressing was created using the hot melt extrusion technique and was capable of releasing metal ions for ~72 hours.

Decellularized small intestine submucosa (SIS) was merged with a mesoporous bioactive glass (MBG) and exosomes using an extrusion-based cryogenic 3D printing process to create a 3D scaffold dressing (SIS/MBG@Exos) that was capable of sustained release of bioactive exosomes.^10^ The 3D structure, adequate porosity, biocompatibility, and hemostasis capabilities of the produced SIS/MBG@Exos hydrogel scaffolds were demonstrated. Hydrogel scaffolds were tested on diabetic wounds, and the results proved their ability to speed up the healing process by boosting blood flow and encouraging the angiogenesis process of the wound.

3D-printed items, in general, do not have desirable mechanical characteristics. Resin homogeneity is another potential difficulty for 3D printing technology. Hydrogel wound dressings can suffer from a loss of mechanical qualities due to fatigue caused by nanoparticles or nanoshaped bubbles in the resin caused by improper mixing of the resin’s constituents. Particularly for patches with nanopores, post-UV and thermal-curing can help with the difficulties. Clinically, the wound dressings are needed to be evaluated as a strong In vitro-in vivo correlation is required to be established. Another major issue with large-scale 3D printing is the cost. The cost of scaling up a lab-based printer and its accessories is so high that it lowers the benefits of 3D printing. However, based on personalized therapies, 3D printing can be a boon to wound treatment.

## Ethical approval

Not applicable.

## Sources of funding

None.

## Author contributions

H.C.: conceptualization, data curation, writing – original draft preparation, writing – reviewing and editing. Priyanka: data curation, writing – original draft preparation, writing – reviewing and editing. O.P.C.: data curation, writing – original draft preparation, writing – reviewing and editing. T.B.E.: writing – reviewing and editing, visualization, supervision.

## Conflicts of interest disclosure

Authors declare that they have no conflicts of interest.

## Research registration unique identifying number (UIN)

Not applicable.

## Guarantor

Talha Bin Emran, PhD, Associate Professor, Department of Pharmacy, BGC Trust University Bangladesh, Chittagong 4381, Bangladesh. Tel: +880 303 356 193, Fax: +880 312 550 224. https://orcid.org/0000-0003-3188-2272

## Data statement

The data in this correspondence article is not sensitive in nature and is accessible in the public domain. The data is therefore available and not of a confidential nature.

## Provenance and peer review

Not commissioned, internally peer-reviewed.
